# Astragaloside IV reduces the expression level of P-glycoprotein in multidrug-resistant human hepatic cancer cell lines

**DOI:** 10.3892/mmr.2014.2074

**Published:** 2014-03-27

**Authors:** PEI-PEI WANG, DU-JUAN XU, CAN HUANG, WEI-PING WANG, WEN-KE XU

**Affiliations:** 1 Department of Pharmacy, Yijishan Affiliated Hospital of Wannan Medical College, Wuhu, Anhui 241001, P.R. China; 2 Department of Pharmacy, The First Affiliated Hospital of Anhui Medical University, Hefei, Anhui 230022, P.R. China; 3 Third-Grade Pharmaceutical Chemistry Laboratory of State Administration of Traditional Chinese Medicine, Anhui Medical University, Hefei, Anhui 230022, P.R. China; 4 Department of Pharmacy, Anqing Shili Hospital, Anqing, Anhui 246003, P.R. China

**Keywords:** astragaloside IV, P-glycoprotein, multidrug resistance, ABCB1

## Abstract

Astragaloside is a saponin widely used in traditional Chinese medicine and has been reported to be a potent multidrug resistance (MDR) reversal agent. The present study investigated the role of astragaloside IV (ASIV) in the regulation of P-glycoprotein (P-gp, encoded by the *mdr1* gene) and its effect on the reversal of MDR. The activity of ASIV was evaluated using human hepatic cancer cells Bel-7402 and the corresponding 5-fluorouracil (5-FU) resistant cells Bel-7402/FU. ASIV (0.08 mg/ml) potentiated the cytotoxicity of 5-FU which was demonstrated using the MTT assay on Bel-7402/FU cells. ASIV reduced the expression of P-gp as was revealed by immunocytochemistry. Accumulation and efflux studies with the P-gp substrate, rhodamine 123 (Rh123), demonstrated that ASIV inhibited P-gp-mediated drug efflux. Furthermore, it was demonstrated that ASIV enhanced the drug accumulation of 5-FU using a high performance liquid chromatography (HPLC) assay for drug resistant cells. Furthermore, ASIV may downregulate the expression of P-gp, which was examined using western blot analysis and polymerase chain reaction. In conclusion, the results of the present study indicated that ASIV reverses the drug resistance of Bel-7402/FU cells by downregulating the expression of *mdr1*. ASIV may represent a potent modulator of P-gp-mediated MDR in hepatic cancer therapy.

## Introduction

Multidrug resistance (MDR) is considered a major cause of failure of anti-cancer chemotherapy. MDR is characterized by the simultaneous resistance to drugs that differ structurally and mechanistically (1). One of the major mechanisms of resistance in MDR mammalian cancer cells involves the increased expression of a 170 kDa transmembrane protein, P-glycoprotein (P-gp). P-gp, a member of the ATP binding cassette (ABC) transporter family, is encoded by *mdr1* genes, also called ABCB1 and works in a similar manner to a pump to extrude anticancer drugs out of cells (2). P-gps expressed in the plasma membrane are mediators of MDR, actively effluxing a wide range of amphiphilic drugs irrespective of concentration gradient, thereby lowering intracellular concentrations to below therapeutic levels (3). The fact that P-gp is overexpressed in various cancer cells has prompted numerous research groups to search for effective inhibitors for this glycoprotein. Several compounds have been proposed as potential MDR modulators, including verapamil, PSC833 and XR9576 (4,5). Verapamil is one of the most extensively tested MDR modulators in the clinic and is used in conjunction with combination chemotherapy strategies. However, there has been limited success due to the cardiac toxicity associated with the high plasma levels required to effectively reverse MDR (6). To date, numerous natural compounds have been demonstrated to be capable of modulating P-gp transport, including rosmarinic acid, glaucine, gypenoside and oroxylin A (7–10).

**Figure 1:**
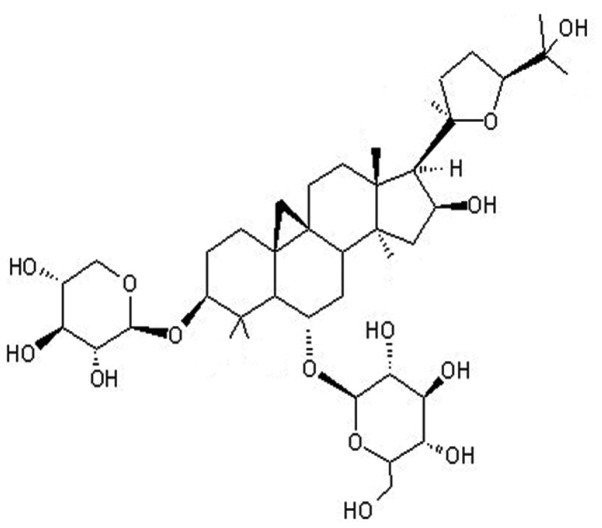
Chemical structure of astragaloside IV.

Radix Astragali [the dried root of *Astragalus membranaceus* (Fisch.) Bunge and *Astragalus mongholicus* Bunge (Fabaceae)] is a nutraceutical commonly used in Traditional Chinese Medicine to treat a variety of diseases (11). It has been reported that Radix Astragali has immunostimulant, cardioprotective and antihyperglycemic effects (12–14). In pharmacopoeia and publications, astragaloside IV (ASIV; a β-D-glucopyranoside with the chemical name (3β,6α,16β,20R,24S)-20,24-epoxy-16,25-dihydroxy-3-(β-D-xylopyranosyloxy)-9,19-cyclolanostan-6-yl) (Fig. 1), is used as a marker for the active constituent in Radix Astragali.

The present study aimed to determine whether ASIV reversed the MDR of the Bel-7402/FU cell line by mechanisms involving the P-gp/*mdr1* gene.

## Materials and methods

### Extraction and isolation of ASIV

ASIV preparation was performed according to a previously published method (15).

### Preparation of ASIV

ASIV was initially dissolved in 70% ethanol and was subsequently dissolved in phosphate buffered saline (PBS) to form a stock solution with a concentration of 4 mg/ml. When the stock solution was used it was diluted to the required concentration with Dulbecco’s modified Eagle medium (DMEM; Gibco, Carlsbad, CA, USA), with the proportion of alcohol in the final concentration <1%.

### Cell culture

The drug-sensitive human hepatic cancer cell line Bel-7402 and the corresponding 5-fluorouracil (5-FU)-resistant Bel-7402/FU cell line were purchased from Keygen Biotech (Nanjing, China). All cells were grown in DMEM (Gibco) supplemented with 10% fetal bovine serum (Gibco) in a CO_2_ incubator. Bel-7402/FU cells were cultured in the previously mentioned medium with addition of 20 μg/ml 5-FU (Tianjin Taihe Pharmaceutical Co., Ltd., Tianjin, China).

### Determination of MDR

Bel-7402 cells and Bel-7402/FU cells were seeded into 96-well plates at 1×10^4^ cells per well. Following 12 h of incubation, cells were treated with various concentrations of 5-FU, mitomycin (Kyowa Hakko Kirin Co., Ltd., Fuji Plant, Shizuoka, Japan) and adriamycin (Actavis Italy S.P.A., Nerviano, Italy) at 0.2, 1, 5, 25 or 125 μg/ml for 48 h. Drug sensitivity was determined by MTT assay according to the manufacturer’s instructions (Sigma-Aldrich, St. Louis, MO, USA). Data were obtained by analyzing the absorption at 550 nm with an automated microplate reader (680; Bio-Rad, Hercules, CA, USA). The IC_50_-values represent the concentrations of the assayed enzymes required to inhibit cell proliferation by 50% and were calculated by using SPSS 13.0 (IBM, Armonk, NY, USA). All reported values are the means of at least three independent experiments. The resistance fold (RF) was calculated by dividing the IC_50_ of resistant cells by the IC_50_ of sensitive cells.

### Determination of cytotoxicity and MDR reversal fold

The *in vitro* cytotoxicity of ASIV was measured by the MTT assay. Bel-7402 and Bel-7402/FU cells were treated with 0.04, 0.08, 0.16, 0.32 or 0.64 mg/ml ASIV for 48 h. The inhibition rate of ASIV on cells was determined using the same MTT assay as described previously.

Bel-7402/FU cells were seeded at 1×10^4^ cells/well and treated with 5-FU (0.025 mg/ml) alone, or in combination with ASIV (0.04 or 0.08 mg/ml) or 0.001 mg/ml (+)-verapamil (purity >99%; Sigma-Aldrich) with 5-FU. Subsequently, the cells were exposed to 5-FU, ASIV or verapamil continuously for 48 h and the cytotoxicity was assessed by MTT assay. The relative reversal fold (RRF) was calculated by dividing the inhibition rate of Bel-7402/FU cells treated with 5-FU and a modulator (ASIV or verapamil) by the inhibition rate of Bel-7402/FU cells treated with 5-FU.

### Immunocytochemistry

The intracellular location and relative expression of P-gp was observed by immunocytochemistry. The cells (5×10^4^/ml) were exposed to 0.08 or 0.16 mg/ml ASIV or 0.001 mg/ml verapamil for 24 h. P-gp was detected using rabbit anti-P-gp monoclonal immunoglobulin G (IgG; Wuhan Boshide Biological Engineering Co., Ltd., Wuhan, Hubei, China) in a 1:200 dilution at 4°C overnight. Subsequently, cells were rinsed three times with phosphate-buffered saline (PBS), and incubated with horseradish peroxidase-conjugated goat anti-rabbit IgG (H+L; Wuhan Boshide Biological Engineering Co., Ltd.) at 1:200 dilution. Cells were observed using fluorescence microscopy (BX51; Olympus, Center Valley, PA, USA).

### Flow cytometric analysis of P-gp function

The cells (5×10^5^/ml) were incubated with or without ASIV (0.08 or 0.16 mg/ml) or verapamil (0.001 mg/ml) for 24 h at 37°C. A total of 10^6^ cells were incubated with 5 μg/ml rhodamine 123 (Rh123) (Sigma-Aldrich) for 1 h at 37°C, washed twice with cold PBS and incubated for 30 min in dye-free medium. Cell fluorescence was evaluated using a flow cytometer (FC500; Beckman Coulter, Miami, FL, USA) at an excitation wavelength of 488 nm and emission wavelength of 525 nm.

### Determination of intracellular drug concentration

Bel-7402/FU cells (5×10^5^/ml) were exposed to 0.025 mg/ml 5-FU in the presence or absence, of 0.08 mg/ml ASIV or 0.001 mg/ml verapamil for 24 h at 37°C. Following trypsinization, the cells were extracted with 500 μl of methanol by ultrasonication and centrifuged at 12,000 × g for 30 min at 4°C. The supernatant was filtered and dried with nitrogen gas. Subsequently, the mobile phase was added to achieve a metered volume of 0.5 ml for the quantitative analysis. Analysis was performed using the Agilent 1100 high performance liquid chromatography (HPLC) system (Agilent Technologies, Santa Clara, CA, USA) comprised of a quaternary pump, an autosampler and a UV detector. A C18 column (250 × 4.6 mm, 5 μm, Diamonsil; Dikma, Lake Forest, CA, USA) was used for the separation. The flow rate was 1 ml/min with methanol:water (10:90, v/v) as the mobile phase. Peak areas were determined at 265 nm for 5-FU.

### Determination of mdr1 mRNA by quantitative polymerase chain reaction (qPCR)

Bel-7402 cells and Bel-7402 cells (5×10^5^/ml) were untreated or treated with 0.08 mg/ml or 0.16 mg/ml ASIV or 0.001 mg/ml verapamil for 24 h. Total RNA was isolated using TRIzol^®^ reagent (Invitrogen Life Technologies, Carlsbad, CA, USA) and qPCR was performed. The primers were as follows: *mdr1* forward, 5′-AAAGTCGGAGTATCTTCTTCCAA-3′ and reverse, 5′-CCAATTTGAATAGCGAAACATTGA-3′); GAPDH forward, 5′-GTGAAGGTCGGTGTCAACGGATTT-3′ and reverse, 5′-CACAGTCTTCTGAGTGGCAGTGAT-3′). PCR conditions were 60 sec at 94°C, followed by 35 cycles of denaturation at 94°C for 60 sec; annealing for 40 sec at 56°C; elongation for 60 sec at 72°C, followed by 10 min at 72°C. There were 35 cycles for *mdr1* and 30 for GAPDH. The total amplification product was subjected to 1.5% agarose gel electrophoresis.

### Western blot analysis

Bel-7402 and Bel-7402/FU cells (5×10^5^/ml) were treated with 0.08 mg/ml or 0.16 mg/ml ASIV or 0.001 mg/ml verapamil. Cells were lysed in a radioimmunoprecipitation assay buffer (Sigma-Aldrich). Cell lysates were boiled at 100°C and cytosolic proteins were separated using 10% SDS-PAGE, transferred onto polyvinylidene fluoride (PVDF) membranes, probed with appropriate antibodies, including goat anti human P-gp monoclonal IgG (Santa Cruz Biotechnology, Inc., Santa Cruz, CA, USA) and horseradish peroxidase-conjugated rabbit anti goat IgG (Santa Cruz Biotechnology, Inc.), and visualized with enhanced chemiluminescence (ECL; Thermo Scientific, Wilmington, DE, USA).

### Statistical analysis

The results are presented as the mean ± standard deviation (n≥3). Statistical analysis was performed with the Student’s t-test. P<0.05 was considered to indicate a statistically significant difference.

## Results

### Determination of MDR

The MTT assay demonstrated that Bel-7402/FU cells were resistant not only to 5-FU but also to adriamycin and mitomycin. Bel-7402/5-FU cells were 19.64-fold more resistant than the control Bel-7402 cells to 5-FU (Table I).

### Cytotoxicity assay of ASIV

**Figure 2:**
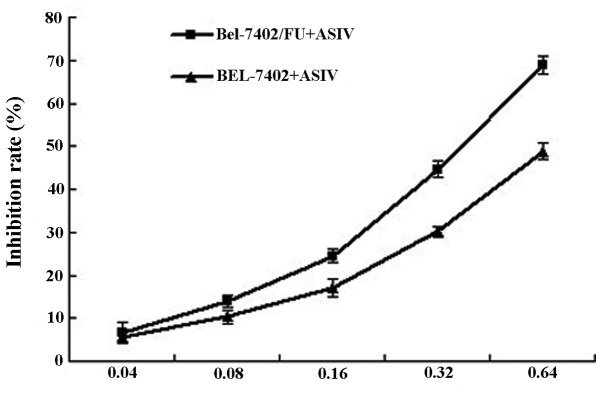
Cytotoxicity of Bel-7402 and Bel-7402/FU cells induced by ASIV. Following 48 h of treatment with ASIV, the inhibition rate of cell viability was assessed using the MTT assay. ASIV, astragaloside IV.

The intrinsic toxicity of ASIV was evaluated in order to ascertain the ability of the modulator to reverse the resistance at nontoxic concentrations. ASIV inhibited the proliferation of Bel-7402 and Bel-7402/FU cells in a dose-dependent manner (Fig. 2). As determined by the dose-effect curve, 0.04 and 0.08 mg/ml of ASIV were not cytotoxic (inhibition rate <5%). Thus, the concentration of 0.04 or 0.08 mg/ml ASIV was used as the dose for the reversal effect of MDR.

### ASIV reverses the resistance of Bel-7402/FU cells to 5-FU

The *in vitro* MDR reversing activity of ASIV was studied by determining the cytotoxicity of 5-FU in Bel-7402/FU cells (Table II). In the presence of 0.08 mg/ml ASIV, the Bel-7402/FU cells exhibited a significantly increased sensitivity to 5-FU. The potency of ASIV was comparable with that of verapamil. The results demonstrated that ASIV was able to reverse MDR *in vitro*.

### ASIV decreases P-gp expression

**Figure 3:**
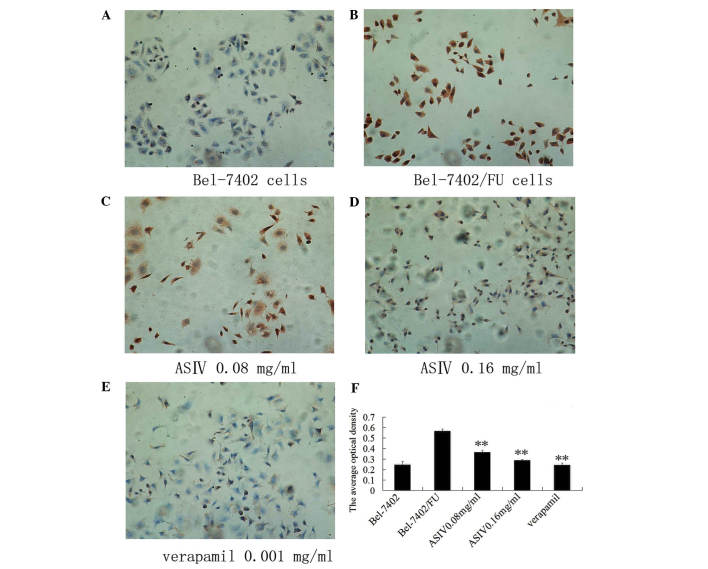
Effect of ASIV on P-glycoprotein by immunocytochemistry assay. Cells were viewed under a fluorescence microscope, magnification, ×400. (A) Bel-7402 cells. (B) Bel-7402/FU cells. (C) Bel-7402/FU cells were treated with 0.08 mg/ml ASIV. (D) Bel-7402/FU cells were treated with 0.16 mg/ml ASIV.(E) Bel-7402/FU cells were treated with 0.001 mg/ml verapamil. Every image was captured by fluorescence microscopy (magnification, ×400) from at least five fields of view. (F) The values of the average optical density were determined using IMAGE-PRO plus image analysis software. Data are the mean ± standard deviation of three independent experiments. ^**^P<0.01, modulator- treated Bel-7402/FU cells vs. untreated Bel-7402/FU cells. ASIV, astragaloside IV.

In order to observe the intracellular location of P-gp and assess the P-gp levels, immuncytochemistry was performed (Fig. 3). Using fluorescence microscopy, the positive response of P-gp was indicated by brown-yellow staining, mainly located in the cytoplasm and cytomembrane, demonstrating a uniform fine granular distribution. As the expression of the Bel-7402/FU group increased, the color darkened. Following the treatment of Bel-7402/FU cells with 0.08 or 0.16 mg/ml ASIV, P-gp expression decreased, the color became lighter and the number of brown-yellow granules in the cytoplasm reduced significantly.

### ASIV decreases the transport activity of P-gp

**Figure 4:**
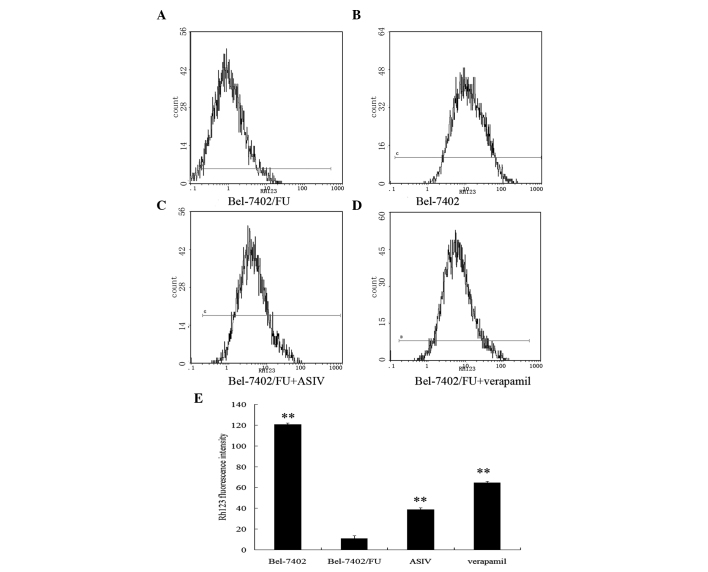
Accumulation and retention of Rh123 in Bel-7402 and Bel-7402/FU cells. The cells were incubated with Rh123 for 60 min prior to being measured by flow cytometry. The intracellular Rh123 concentration was determined. (A) Bel-7402/FU cells; (B) Bel-7402 cells; (C) Bel-7402/FU cells were treated with 0.08 mg/ml ASIV; (D) Bel-7402/FU cells were treated with 0.001 mg/ml verapamil. (E) Rh123 fluorescence intensity. Data are the means ± standard deviation of three independent experiments. ^*^P<0.05, ^**^P<0.01, compared with the Bel-7402/FU cells control group. ASIV, astragaloside IV; Rh123, rhodamine 123.

The ability of ASIV to inhibit P-gp-mediated transport was investigated using the P-gp substrate rhodamine 123 (Rh123). Fig. 4 illustrates that Rh123 accumulation in Bel-7405/FU cells was markedly lower than that found in Bel-7402 cells. Bel-7402/FU cells preincubated with ASIV for 48 h exhibited an increase in the intracellular accumulation of fluorescent Rh123.

### ASIV enhances the intracellular accumulation of 5-FU

**Figure 5:**
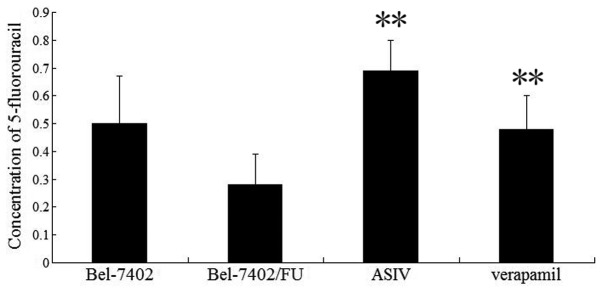
High performance liquid chromatography analysis of the intracellular concentration of 5-FU. The concentration of 5-FU was calculated as the percentage of the total area under the curve for each sample. Intracellular concentrations of 5-FU in untreated control cells were 0.50±0.17 and 0.28±0.11 for Bel-7402 and Bel-7402/FU cells, respectively. Data represent the mean ± standard deviation of three independent experiments. ^**^P<0.01, compared with Bel-7402/FU cells treated with 0.025 mg/ml 5-FU. ASIV, astragalocide IV; 5-FU, 5-fluorouracil.

Intracellular 5-FU accumulation was determined by incubation of Bel-7402/FU cells with 5-FU (0.025 mg/ml) in the presence or absence of (0.08 mg/ml) ASIV by HPLC. Fig. 5 demonstrates that ASIV increased the intracellular accumulation of 5-FU in Bel-7402/FU cells.

### ASIV downregulates mdr1 expression

**Figure 6:**
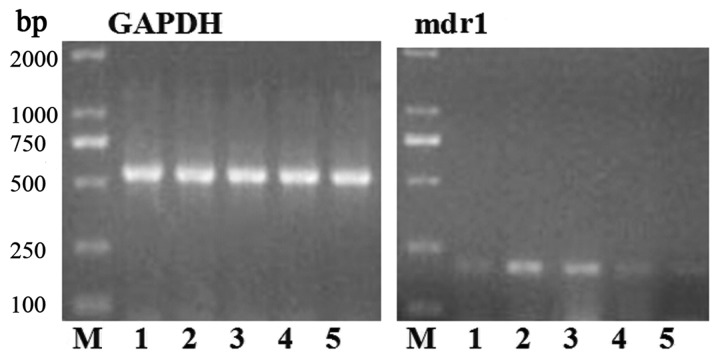

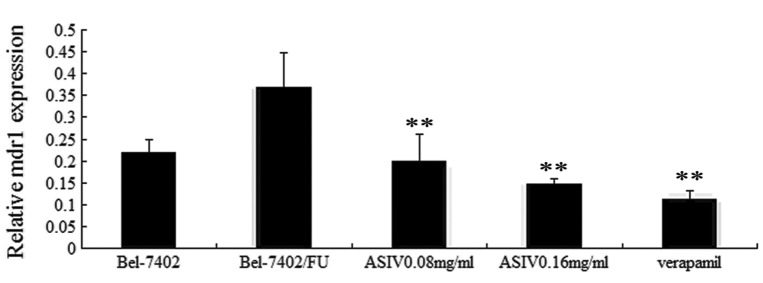
Effect of ASIV on *mdr1* mRNA levels assessed by PCR. The level of GAPDH (PCR product 558 bp) and *mdr1* gene (PCR product 201 bp) were determined. M, Marker DL2000; 1, Bel-7402 cells control; 2, Bel-7402/FU cells control; 3, Bel-7402/FU cells treated with 0.08 mg/ml ASIV; 4, Bel-7402/FU cells treated with 0.16 mg/ml ASIV; 5, Bel-7402/FU cells treated with 0.001 mg/ml verapamil. Relative *mdr1* mRNA levels were calculated by the ratio of *mdr1* densitometric value to the GAPDH densitometric value. Values are presented as the mean ± standard deviation of triplicate experiments. ^**^P<0.01, modulator-treated Bel-7402/FU cells vs. untreated Bel-7402/FU cells. ASIV, astragalocide IV; PCR, polymerase chain reaction; mdr, multidrug resistance.

**Figure 7:**
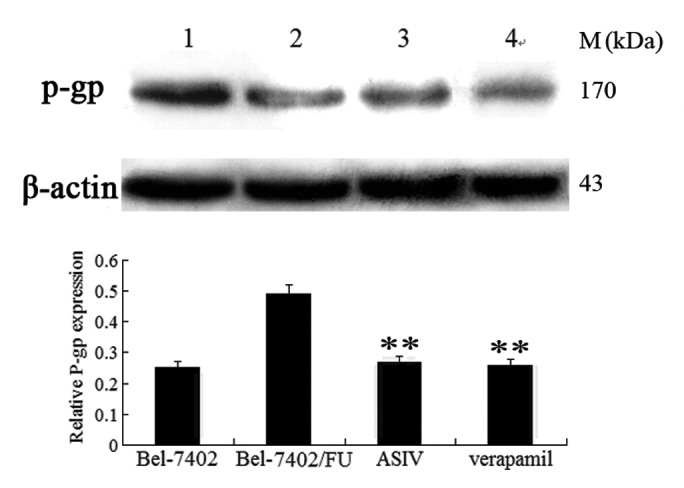
Effect of ASIV on P-gp expression by western blot analysis. 1, Bel-7402/FU cells control; 2, Bel-7402 cells control; 3, Bel-7402/FU cells treated with 0.08 mg/ml ASIV; 4, Bel-7402/FU cells treated with 0.001 mg/ml verapamil. The intensities of the P-gp bands were normalized to β-actin. The ratio of P-gp to β-actin was calculated and expressed relative to that in Bel-7402 and Bel-7402/FU cells. Data are presented as the mean ± standard deviation of three experiments. ^**^P<0.01, modulator-treated Bel-7402/FU cells vs. untreated Bel-7402/FU cells. P-gp, P-glycoprotein; ASIV, astragalocide IV.

Whether ASIV affected *mdr1* mRNA and P-gp expression was examined using qPCR and western blot analysis. As shown in Fig. 6, *mdr1* gene expression was markedly increased in Bel-7402/FU cells compared with Bel-7402 cells. The levels of *mdr1* mRNA were decreased by 0.08 mg/ml ASIV and completely deregulated by 0.16 mg/ml ASIV. As shown in Fig. 7, 0.08 mg/ml ASIV decreased the P-gp levels in Bel-7402/FU cells.

## Discussion

Intrinsic and acquired resistance of malignant cells to cytotoxic agents is a major cause of treatment failure during chemotherapy (16). One of the well-established mechanisms of resistance is the MDR process, due to increased ABC transporter expression (17). Inhibition of drug transporters and modulating MDR are among the most important strategies in the field of cancer chemotherapy.

Previous studies have reported that astragaloside II (ASII) may be capable of reversing hepatoma MDR *in vitro* by downregulating the expression of the *mdr1* gene and P-gp. However, ASII may also inhibit the mitogen-activated protein kinase (MAPK) signal transduction pathway (15). ASIV has been proven to be a novel anti-inflammatory agent and has been suggested as a potential agent for the treatment of cardiovascular diseases (18,19). However, few studies have reported on the effects of ASIV on the reversal of MDR and its molecular mechanisms. In the present study, the *in vitro* potency of ASIV was evaluated with several assays using human hepatic cancer Bel-7402 and Bel-7402/FU cells. Cytotoxicity assays of several anticancer drugs (5-FU, adriamycin and mitomycin) demonstrated that Bel-7402/FU cells were 19.64-fold more resistant to 5-FU than Bel-7402 cells. In order to investigate the reversal effect of ASIV on drug resistant cells, ASIV was administered to cells at the nontoxic concentrations of 0.04 or 0.08 mg/ml, which increased the sensitivity of Bel-7402/FU cells to 5-FU by 1.87-fold. Therefore, ASIV partially reversed the MDR of Bel-7402/FU cells.

P-gp is expressed in a cell- and tissue-specific manner, with high levels detectable in the kidney, liver and intestine (20). The immuncytochemistry assay demonstrated that ASIV significantly inhibited P-gp expression.

The overexpression of P-gp on the surface of tumor cells leads to MDR. This protein acts as an energy-dependent drug efflux pump, reducing the intracellular concentration of structurally unrelated drugs. In the study of accumulation and efflux, sensitive hepatic cancer Bel-7402 cells acted as a negative control and accumulated the most Rh123 compared with Bel-7402/FU cells which accumulated the least (21). However, Bel-7402/FU cells treated with ASIV accumulated more Rh123 than untreated cells. Further confirmation of ASIV inhibiting P-gp-mediated drug efflux was provided by the fact that the modulator increased the intracellular accumulation of 5-FU in Bel-7402/FU cells, according to HPLC analysis. These studies indicated that the reversal of MDR by ASIV was through the enhancement of drug uptake and the inhibition of P-gp mediated drug efflux. Similarly, the reversal of P-gp-mediated MDR with milbemycins correlated with an increase in the accumulation of adriamycin and Rh123 via the inhibition of P-gp efflux in MCF-7/adr cells (22).

The inhibition of P-gp function or inhibition of its expression was able to prevent the P-gp-mediated MDR phenotype and improve the effectiveness of chemotherapy (23).

qPCR and western blot analyses were performed in order to determine the interaction of ASIV with the *mdr1* gene and P-gp. The qPCR assay revealed that ASIV downregulated *mdr1* mRNA expression in Bel-7402/FU cells. Furthermore, western blot analysis revealed that ASIV downregulated P-gp expression in Bel-7402/FU cells. Thus, the downregulation of the *mdr1* gene and P-gp expression by ASIV may be involved in the reversal of MDR.

The MAPK pathway is an important signal transduction pathway activated by various stimuli. Previous reports have demonstrated that modulators of the MAPK pathway may affect the drug transport activity of P-gp in certain multidrug-resistant cell lines (24,25). Adenovirus-mediated enhancement of c-Jun NH_2_-terminal kinase (JNK) reduces the levels of P-gp and reverses P-gp-mediated MDR in human gastric carcinoma resistant cell lines (26). BIRB796, an active inhibitor of p38 MAPK, reverses P-gp-mediated MDR by directly inhibiting its transport function (27). Previous studies have reported that ASII suppressed the phosphorylation of extracellular signal regulated kinase1/2, p38 and the c-Jun NH_2_-terminal kinase. Whether ASIV downregulates P-gp expression via the MAPK pathway requires to be elucidated by future studies.

In conclusion, ASIV has the potential to be used as a P-gp-mediated MDR reversal agent and may be a potential adjunctive agent for human hepatic cancer chemotherapy.

## Figures and Tables

**Table I:** Sensitivity of Bel-7402 and Bel-7402/FU cells treated with chemotherapeutic drugs.

	IC_50_ (μg/ml)		
		
Drugs	Bel-7402/FU	Bel-7402	RF of MDR
5-Fluorouracil	63.35±1.0	3.226±0.3^a^	19.64
Adriamycin	3.259±0.4	1.643±0.5	1.98
Mitomycin	4.428±0.6	2.308±0.7	1.92

IC_50_ is defined as the concentration required to reduce cell proliferation and viability by 50%. All data represent the mean ± standard deviation for at least three independent experiments.

a P<0.01 compared with Bel-7402/FU cells.

RF, resistance fold; MDR, multidrug resistance.

**Table II:** Reversal effects of ASIV on Bel-7402/FU cells.

Drugs	Concentrations (mg/ml)	Inhibition rates (%)	RRF
5-Fluorouracil	0.025	0.129±0.04	-
ASIV + 5-fluorouracil	0.04+0.025	0.209±0.02	1.70±0.43
	0.08+0.025	0.230±0.03^b^	1.86±0.41
Verapamil + 5-fluorouracil	0.001+0.025	0.267±0.03^a^	2.16±0.48

RRF was calculated as the ratio of the inhibition rate of Bel-7402/FU cells treated with a modulator and 5-fluorouracil to the inhibition rate of Bel-7402/FU cells treated with 5-fluorouracil alone. Data are presented as the mean ± standard deviation of three independent experiments compared with Bel-7402/FU cells treated with 5-fluorouracil alone,

a P<0.01;

b P<0.05.

ASIV, astragaloside IV; RRF, relative reversal fold.
